# Supplementary figures and images for: A biome-wide experiment to assess the effects of propagule size and treatment on the survival of Portulacaria afra (spekboom) truncheons planted to restore degraded subtropical thicket of South Africa
Source: PLoS One. 2021 Apr 22;16(4):e0250256. doi: 10.1371/journal.pone.0250256 (PMC8061913; doi:10.1371/journal.pone.0250256)

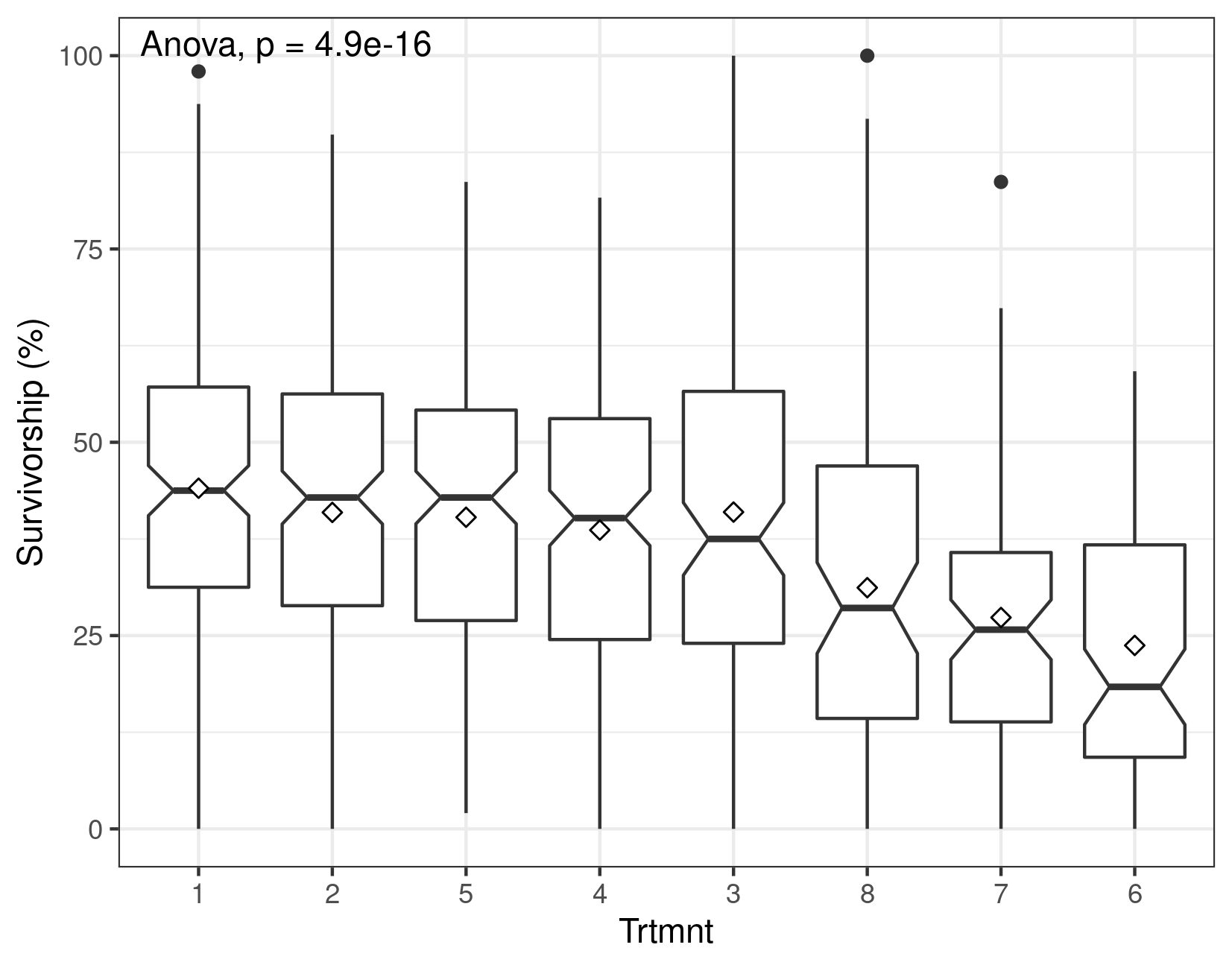

Supplement: S1 Fig — Within-box horizontal lines depict median values and the diamonds depict mean values. (TIF) [file pone.0250256.s001.tif]
